# Quantitative bias analysis for mismeasured variables in health research: a review of software tools

**DOI:** 10.1186/s12874-025-02635-w

**Published:** 2025-08-01

**Authors:** Codie J. C. Gerlach-Wood, Kate Tilling, Jonathan W. Bartlett, Rachael A. Hughes

**Affiliations:** 1 Institute of Statistical Sciences, School of Mathematics, University of Bristol, Woodland Road, Bristol, BS8 1UG UK; 2 MRC Integrative Epidemiology Unit, University of Bristol, Oakfield Road, Bristol, BS8 2BN UK; 3 Population Health Sciences, Bristol Medical School, University of Bristol, Whately Road, Bristol, BS8 2PS UK; 4 Department of Medical Statistics, London School of Hygiene & Tropical Medicine, Keppel Street, WC1E 7HT London, UK

**Keywords:** Quantitative bias analysis, Measurement error, Misclassification, Software tools, Scoping review

## Abstract

**Background:**

Measurement error and misclassification can cause bias or loss of power in epidemiological studies. Software performing quantitative bias analysis (QBA) to assess the sensitivity of results to mismeasurement are available. However, QBA is still not commonly used in practice, partly due to a lack of knowledge of these software implementations. The features and particular use cases of these tools have not been systematically evaluated.

**Methods:**

We reviewed and summarised the latest available software tools for QBA in relation to mismeasured variables in health research. We searched the electronic database Web of Science for studies published between $1^\text {st}$ January 2014 and $1^\text {st}$ May 2024 (inclusive). We included epidemiological studies that described the use of software tools for QBA in relation to mismeasurement. We also searched for tools catalogued on the CRAN archive, in Stata manuals, and via Stata’s *net* command, available from within Stata or from the IDEAS/RePEc database. Tools were included if they were purpose-built, had documentation, and were applicable to epidemiological research. Data on the tools’ features and use cases were then extracted from the full article texts and software documentation.

**Results:**

17 publicly available software tools for QBA were identified, accessible via R, Stata, and online web tools. The tools cover various types of analysis, including regression, contingency tables, mediation analysis, longitudinal analysis, survival analysis and instrumental variable analysis. However, there is a lack of software tools performing QBA for misclassification of categorical variables and measurement error outside of the classical model. Additionally, the existing tools often require specialist knowledge.

**Conclusions:**

Despite the availability of several software tools, there are still gaps in the existing collection of tools that need to be addressed to enable wider usage of QBA in epidemiological studies. Efforts should be made to create new tools to assess multiple mismeasurement scenarios simultaneously, and also to increase the clarity of documentation for existing tools, and provide tutorials and examples for their usage. By doing so, the uptake of QBA techniques in epidemiology can be improved, leading to more accurate and reliable research findings.

**Supplementary Information:**

The online version contains supplementary material available at 10.1186/s12874-025-02635-w.

## Introduction

In epidemiological and population health studies, we often aim to estimate the causal effect of an exposure or treatment on an outcome (referred to as the exposure effect) while adjusting for confounders or other variables [1]. Most methods of estimating an exposure effect rely on the assumption that sufficient confounders are known and have been included in the model, and that the included variables have been measured without error. When data are obtained for epidemiological studies, there is potential for some of the variables to be measured with error and so this assumption may not be plausible [2]. Where we have categorical or binary variables measured with error (as opposed to continuous variables), we refer to measurement error as misclassification. Throughout, the umbrella term “mismeasurement” is used to capture both scenarios.

It is a common misconception that non-differentially mismeasured variables will always bias the effect estimate towards the null [3, 4]. In fact, the impact of mismeasurement on an effect estimate depends on a number of factors, including the role of the variable(s) in which the mismeasurement occurs (i.e., whether it is the outcome, exposure, or other covariate), the type of the variable (i.e., whether it is binary, continuous, or categorical) [5, 6], whether errors in multiple variables are dependent on each other [7], the type of analysis being conducted, and whether the mismeasurement is differential (i.e., some aspect of the error distribution depends on another variable) [8].

Failing to account for mismeasurement can result in problems such as decreased statistical power, biased effect estimates (either towards or away from the null), and inaccurate representations of estimate uncertainty [9]. Any of these issues could result in the reporting of erroneous study conclusions. Inaccurate findings may not only influence government policies and the development of large-scale health interventions, but could also shape the direction of subsequent studies, impact the scientific evidence base, and introduce bias into meta-analyses. Therefore, it is important to account for and quantify the potential effects of mismeasurement. Although potential mismeasurement is sometimes mentioned as a study limitation, it is rarely investigated or adjusted for in practice [4, 10]. A recent review of measurement error in medical literature found that of 565 studies reviewed, only 44% mentioned measurement error at all, with 70% of those doing so only in the discussion section. Of the studies that mentioned mismeasurement, just 7% undertook any investigation or correction [10].

There exist many methods to adjust for mismeasurement, which have been described extensively in the literature [5, 6, 8, 11, 12]. These methods typically require some form of ancillary data, such as validation data (either internal or external), or replication data [9]. However, ancillary data are often not readily available. In these cases, sensitivity analyses such as a quantitative bias analysis (QBA) can be used to evaluate the potential impact of mismeasurement on a study’s conclusions.

QBA consists of a group of statistical methods for assessing uncertainty arising due to biases in a study [13]. It can be applied to various biases, including, but not limited to, unmeasured confounding [14] and selection bias [15]. Here, we focus on QBA for mismeasurement, which is used to quantify the potential impact of mismeasurement or to assess how severe it would need to be to change a study’s conclusions. This allows researchers to assess the robustness of the study’s conclusions to the assumption of no mismeasurement. See Background on quantitative bias analysis section for further information on QBA.

Currently, QBA methods are not employed as standard practice. A recent review found that QBA usage in epidemiology increased between 2006 and 2019 [15], but it was still relatively rare. Possible contributors to the limited use of QBA include the historical lack of available software, limited awareness of existing tools, and the relatively low profile of QBA methods in epidemiological training. Our review seeks to address these challenges by collating information on current software options, highlighting existing gaps, and increasing awareness of the tools available.

There have been several reviews of implementations of QBA methods for unmeasured confounding, misclassification and selection bias in epidemiology and health-related fields [15–20]. These reviews primarily focused on the methodological aspects of QBA—for example, describing available methods, summarising their application in practice, and evaluating their use in empirical studies. They did not review the availability or functionality of software tools used to implement these methods. One review did examine software implementations of QBA methods, but it was specific to tools addressing bias due to unmeasured confounding [14].

In this scoping review, we aim to identify the latest available software tools that implement a QBA for mismeasurement within epidemiological studies quantifying an exposure effect estimate, and provide details on their features and use cases. This will increase awareness of the software and, alongside developments in guidance for researchers on appropriate QBA implementations [19–21], promote its usage as standard practice for health research. We also aim to highlight potential future areas for software development.

### Background on quantitative bias analysis

A QBA for mismeasurement quantifies the likely magnitude and direction of the bias under different plausible assumptions about the mismeasurement process (assuming no other sources of bias). Generally, a QBA requires a model (known as a bias model) for the observed data and the measurement errors [13]. The bias model includes one or more parameters (known as bias or sensitivity parameters), which cannot be estimated from the observed data. These bias parameters encode the researcher’s assumptions about the mismeasurement process, determining the magnitude and direction of the bias-adjustment. For example, in the case of misclassification, the bias parameters may include some combination of the sensitivity, specificity, positive predictive value and negative predictive values^1^. For a continuous variable measured with error, bias parameters may include reliability metrics (such as the reliability ratio or intraclass correlation coefficient), or error quantities (such as error variance or mean squared error).

The observed data alone cannot be used to inform the values of the bias parameters. Researchers must pre-specify values or distributions of values for the bias parameters to enable estimation of the remaining parameters of the bias model and thus obtain a bias-adjusted estimate of the parameter of interest (e.g., the exposure effect). This information is usually obtained from external sources such as validation studies, prior research, expert elicitation, or theoretical constraints [21]. Although the observed data cannot by themselves determine bias parameters, in some circumstances they may provide information to rule out specific combinations of bias parameters as incompatible with the empirical data.

QBA methods can broadly be classified into two categories: deterministic and probabilistic [13]. A deterministic QBA specifies one or more values for each bias parameter. A “simple bias analysis” fixes each bias parameter to a single value (i.e., treating the bias parameter values as known) and outputs a single bias-adjusted estimate of the exposure effect [13].

Typically, the bias parameters are unknown and so the researcher will need to perform a “multidimensional bias analysis” where multiple values are specified for each bias parameter, and the bias model is repeatedly fitted for each combination of bias parameter values. For example, in the case of multidimensional bias analysis for non-differential misclassification of a binary variable, we could consider different pairs of sensitivity and specificity values^2^. A multidimensional bias analysis then outputs multiple bias-adjusted estimates. When there is limited information about plausible values for the bias parameters, a tipping point analysis can be conducted to explore which combinations of values of the bias parameters would overturn study conclusions. This frames QBA not only as a method for estimating the potential impact of bias, but also as a tool for assessing the robustness of study conclusions by identifying how extreme the bias would need to be to meaningfully alter inferences.

In a probabilistic QBA, the researcher specifies a prior probability distribution for each bias parameter. Using this prior distribution, the researcher can specify information about the range of plausible values of the bias parameters, the value combinations that are most likely to occur, and the researcher’s uncertainty about this information. For example, in the case of misclassification, rather than assigning fixed values for sensitivity and specificity as is done in deterministic QBA, the researcher can specify probability distributions (such as Beta distributions) that reflect both plausible ranges and uncertainty. These distributions can differ between cases and non-cases to allow for differential misclassification. Additionally, correlations among parameters (e.g., between sensitivity and specificity for cases) can be incorporated to reflect dependencies. By simulating draws from these joint distributions, the uncertainty in the bias parameters can be propagated through the analysis, yielding adjusted effect estimates with associated uncertainty intervals.

Two main approaches to probabilistic QBA are Bayesian bias analysis (where the prior distribution of the bias parameters is combined with the likelihood function for the data) and Monte Carlo bias analysis (where values of the bias parameters are directly sampled from their distribution and then used to fix the bias parameters to enable estimation of the bias-adjusted exposure effect) [23].

Probabilistic QBA generates an empirical distribution of bias-adjusted effect estimates, which can be summarised using point and interval estimates. The point estimate typically reflects the central tendency (e.g., mean or median) of the distribution under the QBA’s assumptions. The interpretation of the interval estimate depends on the QBA approach: in a Bayesian approach, the interval (a credible interval) can be interpreted as having a specified probability of containing the true exposure effect, conditional on the model and prior. In contrast, in a Monte Carlo approach, the interval (described as a simulation interval [13]) reflects the variability induced by the simulation process and does not necessarily have a direct probabilistic interpretation about the true effect.

A multiple QBA (also known as a multiple-bias analysis) assesses the sensitivity of study results to multiple sources of bias such as mismeasurement, unmeasured confounding, and selection bias. A sequential multiple QBA adjusts for one bias at a time, where the order of adjustment should be based on the reverse order in which the biases occurred during the data generation process [24]. Note that the order of bias adjustments can affect the results of the multiple QBA. When the order of adjustment is in doubt, the researcher should assess sensitivity of the conclusions of the multiple QBA to different ordering of the bias adjustments [13]. A simultaneous multiple QBA avoids this issue because it adjusts for the multiple sources of bias simultaneously [25].

## Methods

We searched for QBA software described in health research articles, as well as those available in software databases, published between $1^\text {st}$ January 2014 and $1^\text {st}$ May 2024 (inclusive). We selected this 10-year time frame to focus on recent tools that are methodologically current and more likely to be actively maintained.

We define a QBA as a method that adjusts for mismeasurement using a model that includes one or more bias parameters. Also, “software” is defined as a web tool, package, or code that is publicly available to use, is not specific to a particular data example, and is accompanied by documentation. To be classified as having “documentation”, a tool must provide enough information for users to understand its function and implementation without reliance on an external publication. This includes a user guide or in-code comments that explain the syntax, input requirements, and expected outputs. Examples of tools not meeting our software definition would be tools that were not publicly available, code files for specific examples that the researcher had to manually edit to apply to their study, and software that lacked documentation describing how to use the code, such as raw code files without explanatory comments.

We searched published literature from health research and software databases, as this is how most health researchers would identify methods and tools they can use. We did not look for methods within textbooks as these are not publicly available and often cannot be easily searched by applied researchers.

This review was written following the Preferred Reporting Items for Systematic reviews and Meta-Analyses extension for Scoping Reviews (PRISMA-ScR) guidelines [26], and the PRISMA-ScR checklist can be found in Additional File 1. Our search was conducted in three steps: search implementation, eligibility screening and data extraction.

### Publication search

**Fig. 1:**
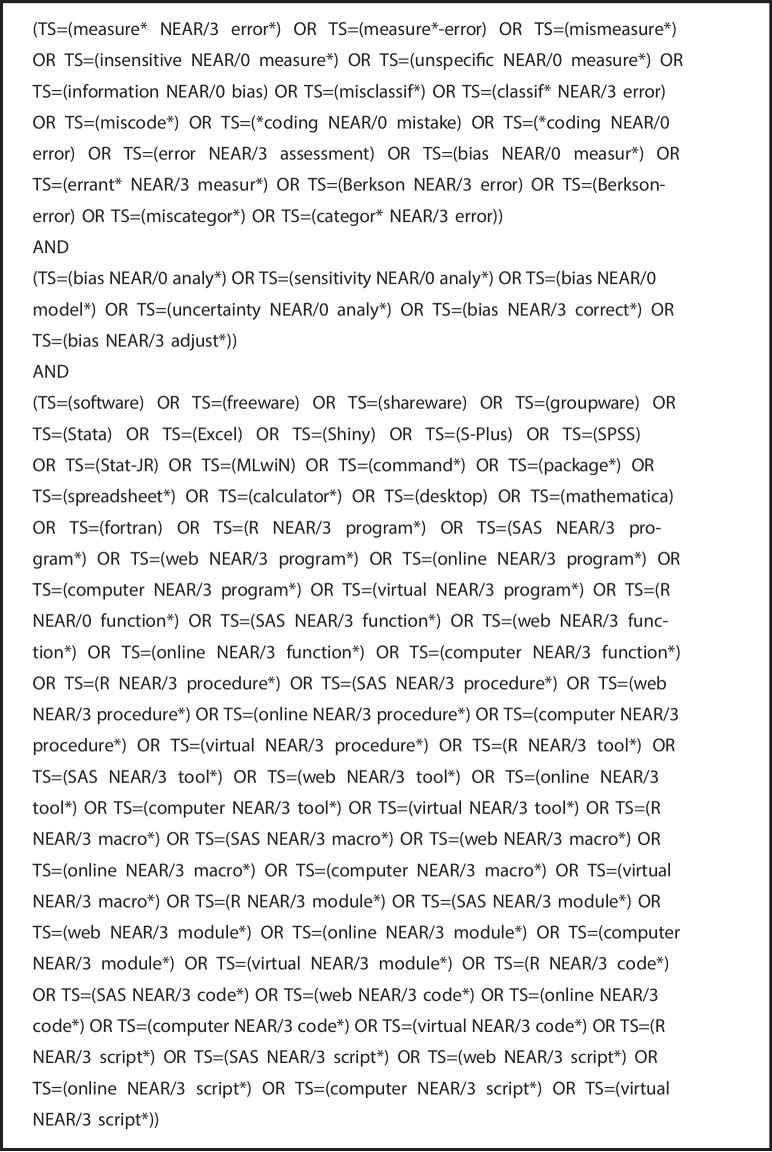
Clarviate Web of Science search terms for this software review [27]

**Fig. 2:**
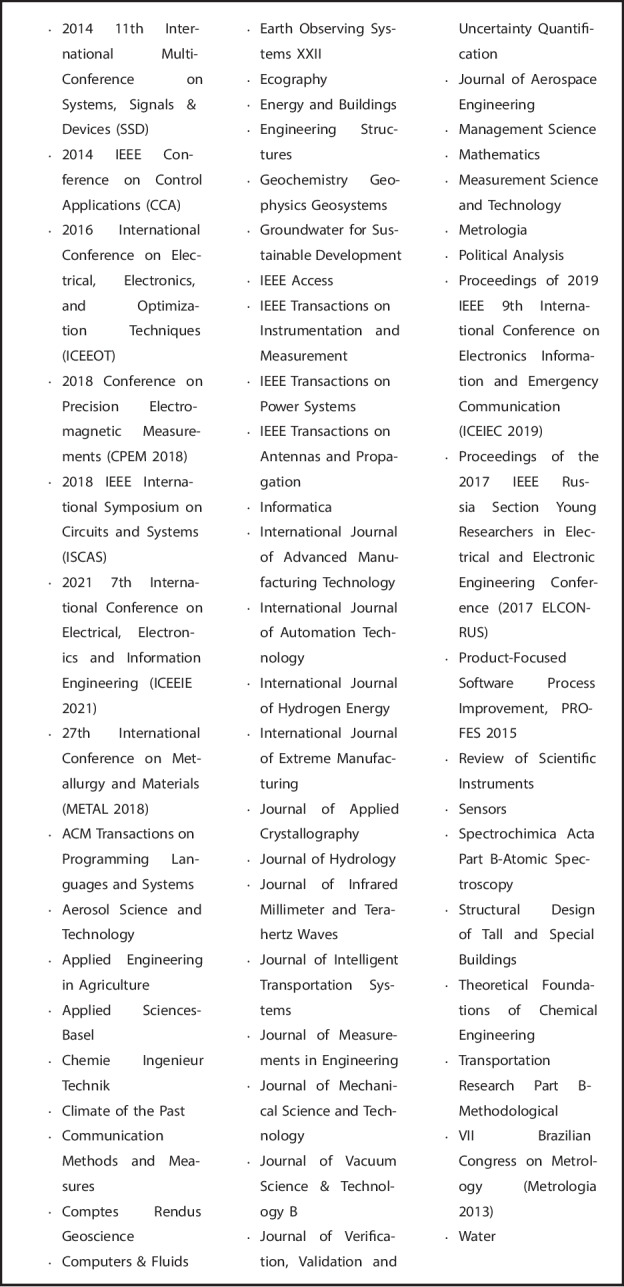
Journals outside of the scope of health research that were removed from our search results

In our first step, we used Web of Science to identify papers that mentioned all of the terms “measurement error”, “bias analysis” and “software” (or some other synonym of these terms) in either the title, abstract or as keywords. The specific search terms used can be seen in Fig. 1 [27]. The search was applied to databases “Web of Science Core Collection”, “BIOSIS Citation Index”, “KCI-Korean Journal Database”, “MEDLINE” and “SciELO Citation Index”. We excluded from our search any meeting abstracts, clinical trials or patents. In addition, we excluded any articles published in journals outside of the fields of statistics, medicine, population health, and epidemiology and so deemed out of the scope of health research. A list of the excluded journals is given in Fig. 2.

### Software repository search

In order to capture software implementations not mentioned in published literature, we completed several additional searches outside of Web of Science. We searched The Comprehensive R Archive Network (CRAN) [28], R’s central software repository containing a large collection of quality-assured contributed packages. We also searched IDEAS/RePEc [29], an online database indexing items of economics research including articles as well as Boston College’s Statistical Software Components (SSC) archive, which contains user-written Stata commands and other code. In addition, using Stata’s *search* command, we conducted a search of the Stata manuals, the Stata Journal, and all Stata-related user-written commands that are available via Stata’s *net* command.

We limited our results to tools which were first made publicly available (or had updated versions with new features implementing a QBA to mismeasurement) between $1^\text {st}$ January 2014 and $1^\text {st}$ May 2024 (inclusive). To implement this criterion, we manually checked the publication or update date of each tool and excluded any that fell outside the specified period.

#### CRAN

For our search of CRAN, we identified packages that mentioned both “measurement error” and “bias analysis” (or synonyms of these terms) in either their title or description. The R code used to implement this search is included in Additional File 2.

We first used R’s built-in CRAN package repository tools to extract the names, titles and descriptions of all of the packages maintained on CRAN on the search date, $8^\text {th}$ May 2024. After cleaning the extracted text, removing new line breaks and any multiple spaces, we then used the R *grep* function, which searches for pattern matches to its argument, to search for those packages which mentioned both “measurement error” and “bias analysis” in their title or description. Search terms and synonyms used were equivalent to those in Fig. 1, in order to maintain consistency between our publication search and our repository search.

#### IDEAS/RePEc and Stata

**Fig. 3:**
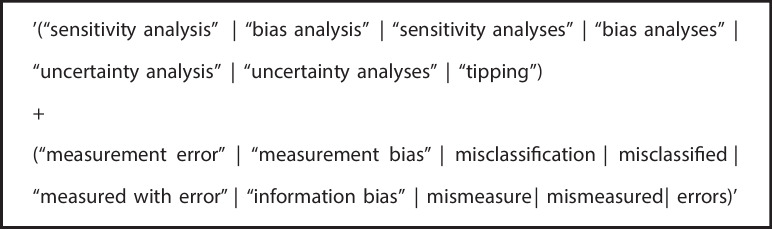
IDEAS/RePEc and Stata manual search terms for this software review

The “advanced search” tool of IDEAS was less flexible than the R functions used to search CRAN, and so for this database we simplified our search strategy. We searched IDEAS for software components that had both “measurement error” and “bias analysis” or their synonyms in any of their title, abstract or key words using the search string given in Fig. 3. We also used this same set of terms to search the Stata manuals, the implementation of which can be found in Additional File 3. We considered all results which referenced either “measurement error” or “bias analysis” or their synonyms.

### Eligibility criteria

In our second step, the eligibility of identified abstracts and tools was assessed independently by two reviewers (CGW and RH), with any disagreements resolved by consensus. Abstracts and tools were eligible for data extraction if they satisfied all of the following criteria:

1. the abstract mentioned purpose-built software,
2. the abstract discussed bias due to mismeasurement,
3. the software implemented a QBA for mismeasurement.

Examples of abstracts that would be excluded were those that only mentioned programming languages or code for examples rather than providing a purpose-built tool, abstracts where a QBA was not conducted but mentioned as further work, and abstracts which had software for purposes other than a QBA for mismeasurement.

### Data extraction

In our third step, we examined the full texts of the included published papers and the documentation of the packages found via our CRAN, Stata manual, Stata *net* command and IDEAS/RePEc database searches to extract information about any software presented and its features. We excluded any R packages that had been removed from CRAN, software that could not be loaded, and software with example code that failed to run due to unhandled errors. We also excluded any sensitivity analysis implementations that did not meet our definition for software or could not be considered a QBA due to not including at least one bias parameter.

**Fig. 4:**
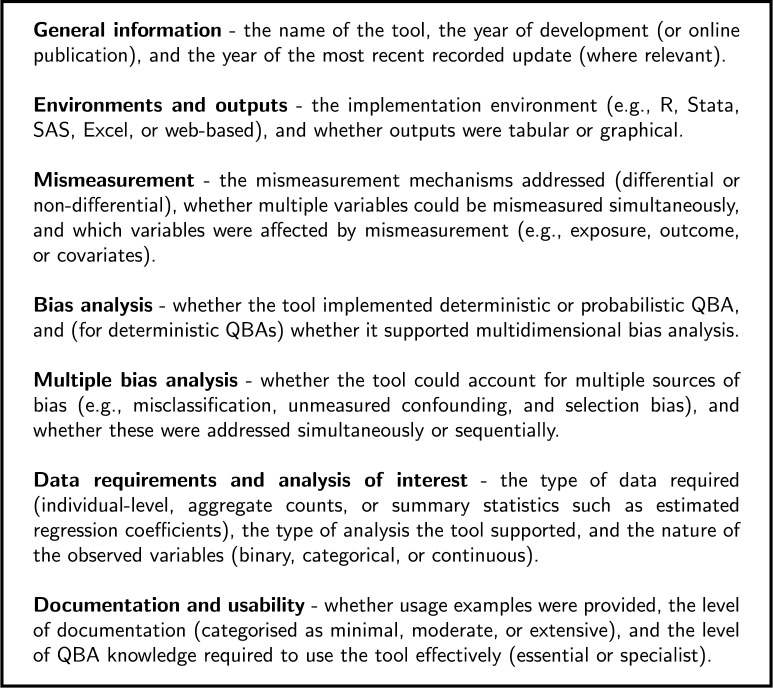
Domains of data extraction for each software tool

Information was extracted on several data domains, reflecting tool characteristics and capabilities. An overview and description of the data collected is given in Fig. 4.

When assessing the documentation and usability of the tools, two reviewers (CGW and RH) independently evaluated the level of detail in the documentation and the level of QBA knowledge required to perform a multidimensional or probabilistic QBA using the tool. Any disagreements were resolved by consensus.

Documentation was categorised into three levels; minimal, moderate and extensive. Tools classified as having minimal documentation provided only a brief description of the tool’s purpose, required inputs, and syntax (where applicable). Moderate documentation included a full description of each function, at least one usage example, a written explanation of the output, and a detailed description of the method implemented. Tools at this level also provided practice datasets where applicable. Tools classified as having extensive documentation offered additional tutorial materials such as vignettes, video tutorials, or an accompanying software journal article.

The level of QBA knowledge required was classified as either essential or specialist. Requiring essential knowledge indicated that the tool fully implemented a multidimensional or probabilistic bias analysis and displayed the results without researchers having to manually code these steps. Specialist knowledge was deemed to be required when researchers had to manually implement or visualize a multidimensional or probabilistic bias analysis. Alternatively, the tool may have required expertise in Bayesian methods, such as defining priors or assessing the convergence of MCMC samplers.

## Results

### Publication search

**Fig. 5:**
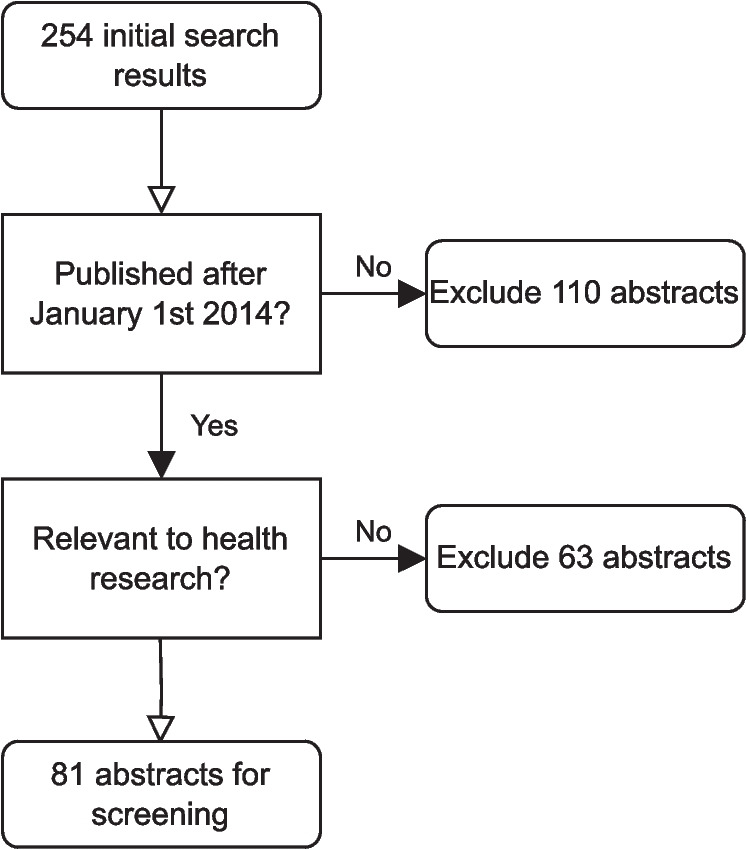
Flowchart of the publication search step of the review

After removal of duplicates, our initial Web of Science search returned 254 results. We then excluded 110 papers when restricting to publications made between $1^\text {st}$ January 2014 and $1^\text {st}$ May 2024. A further 63 papers were manually excluded that were published in journals outside of the scope of health research (as listed in Fig. 2). We were left with a total of 81 abstracts. This initial search step is illustrated in Fig. 5.

**Fig. 6:**
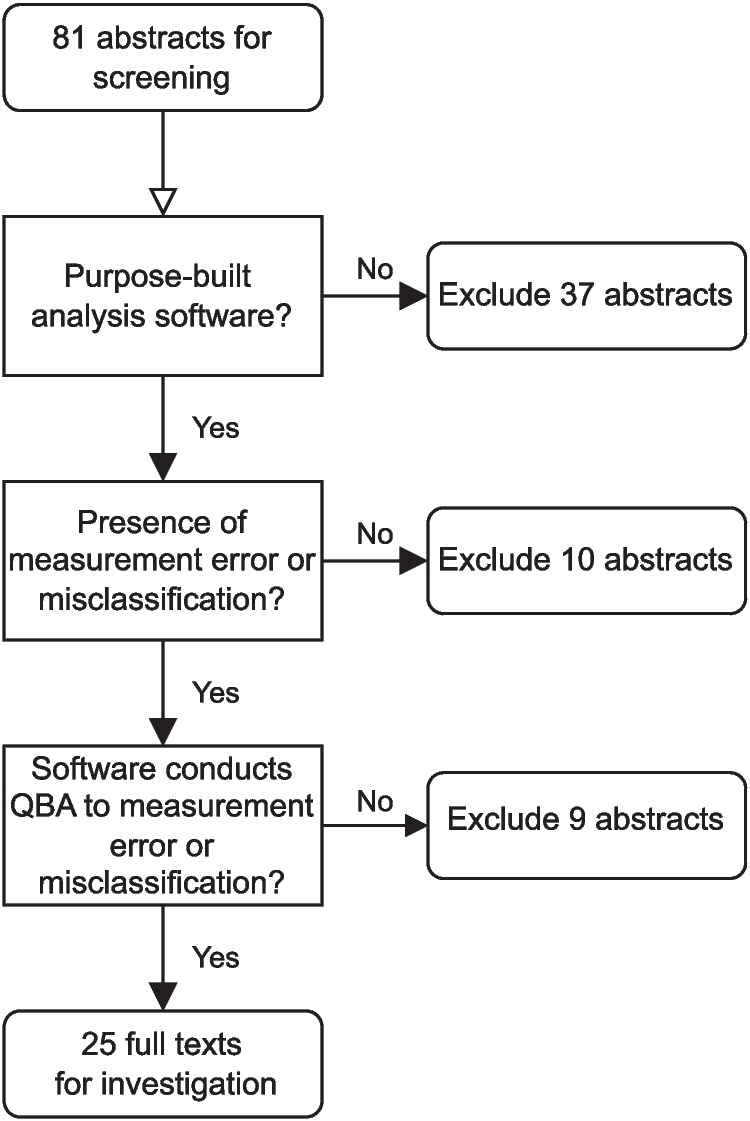
Flowchart of the abstract screening step of the review

We excluded 37 abstracts which did not provide a purpose-built statistical software implementation, 10 abstracts that did not focus on bias due to mismeasurement, and nine abstracts where the software provided was not conducting a QBA for mismeasurement (e.g., the QBA was instead for an alternative form of bias). The abstract screening process is illustrated in Fig. 6. When reviewing the full text of the remaining 25 articles, we found references to 24 unique software tools.

### Software repository search

Our IDEAS search identified a single Stata command, *episens* [30, 31], however, the mismeasurement-related functions of this tool had not been updated since 2008, and so the tool fell outside of our date range for eligibility. We discuss this tool further in Grey literature section, and in Table 2.

Our CRAN search returned ten software tools, four of which had also been identified by our Web of Science search. The tool *multibias* [32] also referenced an additional web tool implementation in its documentation, *multibias web tool* [33]. Thus, in total, our CRAN search provided an additional seven tools.

Our search using the Stata *search* command returned an initial 205 results, 136 of which were either duplicates or were outside of our date range and so were excluded. From the 69 remaining search results, four eligible tools were found.

### Eligibility criteria

**Fig. 7:**
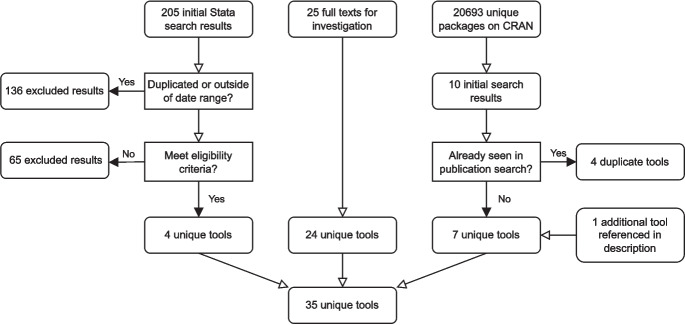
Flowchart of the software tool search process

In total, 35 unique software tools were identified across all of our searches, the process of which is summarised in Fig. 7. Among these 35 tools, nine were excluded because they did not implement a QBA to mismeasurement, four were excluded because they did not provide sufficient documentation or were code for a specific example requiring user adjustment^3^, one tool was excluded because it failed to run example code due to an unresolved runtime error at the time of review, and one tool was excluded because it had been removed from CRAN. The remaining 20 met our inclusion criteria.

Of these 20 tools, web tool *APScalculator* [36], Stata command *bamm* [37], and R package *SensiPhy* [38] do not implement a QBA for an exposure effect estimate of an epidemiological study and so are excluded from the results presented in Table 1. The web tool *APScalculator* evaluates the impact of classical measurement error ([6], Chapter 1) on the categorization of a continuous variable into user-specified groups, rather than directly assessing bias in effect estimates. The R package *SensiPhy* estimates the impact of various sources of uncertainty in phylogenetic comparative methods used within ecology, which differs substantially from applications in health research. The Stata command *bamm* conducts a Bayesian bias analysis to investigate the distribution of a single misclassified categorical variable, which could be either nominal or ordinal.

**Table 1:** Software programs implementing a quantitative bias analysis for mismeasurement published between 2014 and 2024

Software name (Year)	Environment	Output		Mismeasurement			Bias analysis	
		Table	Plot	Type	Differential	Multiple mismeasured variables	Type	Multi dimensional
*biasepi* (2019) [39, 40]	Stata command	✓	-	MC	D, ND	-	Det	-
*pvw* (2019) [41]	Stata command	✓	-	MC	D, ND	-	Det	-
*SAMBA-EHR* (2020) [42, 43]	Web tool	-	✓	MC	ND	-	Det	✓
*Outcome Misclassification* (2020) [44]	Web tool	✓	✓	MC	D, ND	-	Det	✓
*SensitivityAnalysis* (2020) [45]	Web tool	-	✓	MC	ND	-	Prob	-
*miCoPTCM* (2016, upd. 2020) [46]	R package	✓	-	ME	ND	✓	Det	-
*ivbounds* (2021) [47, 48]	Stata command	✓	-	MC	ND	-	Det	✓
*MediationSensitivityAnalysis* (2021) [49]	Web tool	✓	✓	ME	ND	✓	Det	✓
*BayesSenMC* (2021) [50]	R package	✓	✓	MC	D, ND	-	Prob	-
*EValue* (2017, upd. 2021) [51, 52]	R package	✓	-	MC	D	✓	Det	-
*ConMed* (2023) [53]	R package: non-CRAN	-	✓	ME	ND	✓	Det	-
*episensr* (2015, upd. 2023) [54]	R package	✓	✓	MC	D, ND	✓	Det, Prob	✓
*apisensr* (2021, upd. 2023) [55, 56]	Web tool	✓	✓	MC	D, ND	-	Det, Prob	✓
*mgee2* (2020, upd. 2023) [57, 58]	R package	✓	✓	MC	ND	✓	Det	-
*multibias web tool* (2023) [25, 33]	Web tool	✓	✓	MC	D, ND	-	Det, Prob	-
*multibias* (2023, upd. 2024) [25, 32]	R package	✓	-	MC	D, ND	✓	Det	-
*rcme* (2023, upd. 2024) [59]	R package: non-CRAN	✓	✓	ME	D, ND	-	Det	-

Software name (Year)	Analysis				
	Data type	Analysis of interest	Outcome	Exposure	Other covariates
*biasepi* (2019) [39, 40]	Individual, Aggregate	Contingency table	**bin**	**bin**	-
*pvw* (2019) [41]	Individual	Regression	bin	**bin**	bin, cat, cts
*SAMBA-EHR* (2020) [42, 43]	Summary	Regression	**bin**	cts, cat	-
*Outcome Misclassification* (2020) [44]	Summary	Contingency table	**bin**	bin	-
*SensitivityAnalysis* (2020) [45]	Summary	Regression	cts	bin	**bin**
*miCoPTCM* (2016, upd. 2020) [46]	Individual	Survival analysis	TTE	**cts**	**cts**
*ivbounds* (2021) [47, 48]	Individual	Instrumental variable analysis	bin, cat, cts	**bin**	bin, cat, cts
*MediationSensitivityAnalysis* (2021) [49]	Summary	Mediation analysis	**cts**	bin	**Mediator (cts)**, **other (cts)**
*BayesSenMC* (2021) [50]	Aggregate	Contingency table	bin	**bin**	-
*EValue* (2017, upd. 2021) [51, 52]	Summary	Regression	**bin**	**bin**	-
*ConMed* (2023) [53]	Summary	Mediation analysis	**cts**	cts	**Mediator (cts)**, other (bin, cat, cts)
*episensr* (2015, upd. 2023) [54]	Aggregate	Contingency table	**bin**, TTE	**bin**	**bin**
*apisensr* (2021, upd. 2023) [55, 56]	Aggregate	Contingency table	**bin**	**bin**	**bin**
*mgee2* (2020, upd. 2023) [57, 58]	Individual	Longitudinal analysis	**cat (ordinal)**	**cat (ordinal)**, bin, cts	**cat (ordinal)**, bin, cat, cts
*multibias web tool* (2023) [25, 33]	Individual	Regression	bin	**bin**	bin, cat, cts
*multibias* (2023, upd. 2024) [25, 32]	Individual	Regression	**bin**	**bin**	bin, cat, cts
*rcme* (2023, upd. 2024) [59]	Individual	Regression	**cts**	**cts**	**cts**

Software name (Year)	Usage		
	Level of documentation	Examples	QBA knowledge required
*biasepi* (2019) [39, 40]	Moderate	✓	Specialist
*pvw* (2019) [41]	Moderate	✓	Specialist
*SAMBA-EHR* (2020) [42, 43]	Extensive	✓	Essential
*Outcome Misclassification* (2020) [44]	Extensive	✓	Essential
*SensitivityAnalysis* (2020) [45]	Moderate	✓	Essential
*miCoPTCM* (2016, upd. 2020) [46]	Moderate	✓	Specialist
*ivbounds* (2021) [47, 48]	Extensive	✓	Essential
*MediationSensitivityAnalysis* (2021) [49]	Minimal	-	Essential
*BayesSenMC* (2021) [50]	Extensive	✓	Specialist
*EValue* (2017, upd. 2021) [51, 52]	Extensive	✓	Essential
*ConMed* (2023) [53]	Moderate	✓	Essential
*episensr* (2015, upd. 2023) [54]	Extensive	✓	Essential
*apisensr* (2021, upd. 2023) [55, 56]	Extensive	✓	Essential
*mgee2* (2020, upd. 2023) [57, 58]	Moderate	✓	Specialist
*multibias web tool* (2023) [25, 33]	Minimal	-	Essential
*multibias* (2023, upd. 2024) [25, 32]	Extensive	✓	Specialist
*rcme* (2023, upd. 2024) [59]	Minimal	✓	Specialist

**Bold text** for the “Outcome”, “Exposure” and “Other covariates” columns indicates the variable can be considered mismeasured by the tool. “Aggregate” data here means any non-individual level data e.g., count data. “Summary” data here means statistics calculated from data, e.g., regression coefficients or standard deviations

Abbreviations used (in alphabetical order): *D* Differential, *Det* Deterministic, *MC* Misclassification, *ME* Measurement error, *ND* Non-differential, *Prob* Probabilistic, *bin* Binary, *cat* Categorical, *cts* Continuous, *TTE* Time-to-event, *upd.* Updated

### Overview of included tools

Table 1 summarises the key features of the 17 total software programs we found that are applicable to health studies aiming to quantify bias in an effect estimate, in order of most recent update.

#### Environments and outputs

Of the tools reviewed, eight (47%) are implemented as R packages, six (35%) are web-based applications, and three (18%) are Stata commands.^4^

Approximately half of the tools (eight) provide both graphical and tabular outputs to aid the researcher in interpreting the results. Only three tools do not produce tables: web tool *SAMBA-EHR*, web tool *SensitivityAnalysis* and R package *ConMed*. Three R packages and three Stata commands do not provide graphical plots of their results.

#### Mismeasurement

Of the 17 tools, four implement a QBA in the case of measurement error in a continuous variable (R packages* miCoPTCM*,* ConMed* and *rcme*, and web tool *MediationSensitivityAnalysis*). Only R package *rcme* allows for multiplicative measurement error (i.e., error that scales with the true value of the variable), whilst the rest of the tools employ a classical additive measurement error model ([6], Chapter 1). A total of 12 tools apply a QBA for misclassification of a binary variable. Only one tool, R package *mgee2*, implements a QBA when the misclassified variable has more than two categories.

In total, 11 software tools handle cases of outcome mismeasurement, 12 handle exposure mismeasurement, and seven handle mismeasurement in other covariates (such as effect modifiers, mediators, or potential confounders). Among these, two tools (web tools *SAMBA-EHR* and *Outcome Misclassification*) focus solely on outcome mismeasurement, four (Stata commands *pvw* and *ivbounds*, R package *BayesSenMC*, and *multibias web tool*) exclusively handle exposure mismeasurement, and only the web tool *SensitivityAnalysis* is specific to misclassification of a confounder.

Among the tools, nine are applicable for both differential and non-differential mismeasurement, while seven are for non-differential mismeasurement only. The R package *EValue* is specific to differential misclassification. Multiple variables can be mismeasured simultaneously in seven (41%) of the tools.

#### Bias analysis

A deterministic QBA is implemented exclusively in 11 tools, two (web tool *SensitivityAnalysis* and R package *BayesSensMC*) support only a probabilistic QBA, and three include options to implement both a deterministic and probabilistic QBA (R package *episensr*, web tool *apisensr*, and *multibias web tool*^5^). Among the tools that implement a probabilistic QBA, only R package *BayesSenMC* performs a Bayesian bias analysis, and the remaining tools perform a Monte Carlo bias analysis. Among the tools that implement a deterministic QBA, only six (40%) perform a multidimensional analysis.

#### Multiple bias analysis

The Stata command *biasepi*, web tools *MediationSensitivityAnalysis* and *multibias web tool*, and the R packages *EValue*, *ConMed*, *episensr*, and *multibias* can all perform a multiple QBA for mismeasurement and unmeasured confounding. Additionally, Stata command *biasepi*, R package *EValue*, R package *multibias*, and *multibias web tool* can also adjust for selection bias. Note that all tools except R package *EValue*, R package *episensr*, and Stata command *biasepi* adjust for multiple sources of bias simultaneously; these tools instead apply a sequential approach.

#### Data requirements and analysis of interest

All tools except Stata command *biasepi*, which can take either individual or aggregate data as inputs, are specific in the data type required. Individual-level data is required by seven of the tools, aggregated count data by four tools, and summary statistics (such as regression coefficients or other statistics derived from the data) are required by six of the tools.

When the analysis of interest is a mediation analysis, two tools are applicable: web tool *MediationSensitivityAnalysis* and R package *ConMed*. The web tool *MediationSensitivityAnalysis* performs bias analysis for measurement error in the outcome, mediator, or other observed covariates (effect modifiers or potential confounders), with the assumption that the binary exposure is measured without error. The R package *ConMed* adjusts for measurement error in the mediator or outcome, and is applicable specifically when there is unmeasured confounding as well as measurement error.

For other types of analysis of interest, R package *miCoPTCM* accounts for measurement error of a continuous covariate or exposure in survival analysis (specifically a promotion time cure model), where the outcome is a time-to-event variable. Stata command *ivbounds* applies QBA for an instrumental variable analysis, allowing for a binary or categorical instrumental variable. R package *mgee2* conducts QBA for a longitudinal analysis, where there are changes within the same individuals or groups over time. Of the remaining tools, five are applicable for the analysis of contingency tables and seven for logistic or linear regression.

Most tools require the outcome variable of the analysis of interest to be either binary (nine programs) or continuous (five programs). Only R package *mgee2* and Stata command *ivbounds* allow for a discrete outcome variable with more than two categories. The exposure variable of the analysis of interest is typically required to be exclusively either binary (nine programs) or continuous (four programs), but two programs (web tool *SAMBA-EHR* and R package *mgee2*) allow exposure variables to be of multiple types including both discrete or continuous options. Of all of the tools, 12 (71%) allow for the inclusion of other covariates than just the exposure and outcome in the analysis.

#### Documentation and usability

Of the 17 tools reviewed, only two do not include usage examples, both of which are web tools. Documentation quality varies: eight tools have extensive documentation, six have moderate documentation, and three have minimal documentation. Ten tools fully implement a multidimensional or probabilistic QBA (i.e., user only requires essential QBA knowledge as the software implements all steps of the QBA including summaries of the results). However, seven tools require users to have specialist knowledge (e.g., a tool only performs a simple bias analysis and so a user must write their own code to conduct a probabilistic QBA using this tool).

### Grey literature

In our formal search we focused on software described in the published literature between January $1^\text {st}$ 2014 and May $1^\text {st}$ 2024, or software made available during this period via CRAN, the Stata manuals, the IDEAS/RePEc database, or other Stata user-written commands available using Stata’s *net* command. This approach ensured we captured the latest tools which applied researchers could readily locate. However, we recognise that additional software exists that was not identified through this search. For example, some tools have not been mentioned in journal articles and are hosted in alternative environments such as web-based platforms. Others were developed before 2014 and have not been significantly updated since but remain in use. Table 2 gives a non-exhaustive brief overview of some tools known to the authors that were not captured by our formal search strategy, but that may be of interest to researchers.

**Table 2:** Software tools for mismeasurement correction and QBA not captured by our formal search strategy

Tool	Environment	Brief description
*Quantitative Bias Analysis* [64]	Web tool	Conducts simple and multidimensional bias analyses for misclassification and unmeasured confounding.
*CMAverse* [65, 66]	R package (non-CRAN)	Conducts sensitivity analyses for unmeasured confounding, measurement error, and selection bias in causal mediation analysis.
*episens* [30, 31]	Stata	Provides basic sensitivity analysis of the observed relative risks, adjusting for unmeasured confounding and misclassification of the exposure.
*eivtools* [67]	R package	Functions for analysis with error-prone covariates.
*Prob Bias Analysis for Information Bias* [68]	Web tool	Probabilistic bias analysis for misclassification; an R based web interface for *episensr* [54].
*simplemba* [69]	Web tool	Odds ratio calculator with misclassification, selection bias, and confounding adjustment.
*sensmac* [70, 71]	SAS	Implements probabilistic sensitivity analysis to misclassification of a binary variable.
*Multiple bias model* [72, 73]	SAS	Code and dataset for conducting multiple bias modelling.
*Mecor* [74]	R package	Functions to perform covariate measurement error correction.
*Short code* [34, 35]	R and SAS code	Code to perform record and summary level QBA, for misclassification and confounding.
*Misclassification spreadsheet* [13, 75]	Excel spreadsheet	Spreadsheet to perform simple bias analysis for misclassification in contingency tables.

*Short code* [34, 35], which consists of SAS and R code, is included in Table 2 despite having been initially captured in our formal search and later excluded based on our inclusion criteria; specifically, due to a lack of adequate documentation (see Eligibility criteria section). It is the only tool in Table 2 that passed initial screening stages but was actively excluded from the final review; the rest were not captured by our formal search. We include it here due to its prominence in the QBA literature and its potential value for learners, particularly when used alongside [13].

Another notable inclusion in Table 2 is the *Misclassification spreadsheet* Excel tool [75], which accompanies [13]. Although the spreadsheet was not captured by our formal search due to its distribution via a textbook website rather than publication or software repositories, we note that it has been widely disseminated (including, since this review was conducted, being directly linked in methodological guidance [76]). This spreadsheet remains a valuable and accessible tool for introducing researchers to the mechanics of QBA. Similarly, the *episens* command for Stata, originally released in 2008 [30], remains relatively widely used in the Stata community for QBA to exposure misclassification.

## Discussion

We have conducted an up-to-date review of software implementations of QBA to mismeasurement described in the published literature, R packages available on CRAN, and Stata commands, including user-written commands available from the SSC archive or via Stata’s *net* command. All software tools were developed or significantly updated post-2019, with most (65%) having been developed or updated since 2021. The software tools were either R packages, Stata commands or online web tools and were available for routine analyses of interest such as linear regression and contingency tables, and for more specialized analyses such as mediation analysis, instrumental variable analysis and survival analysis. All but one software tool implemented a QBA to non-differential mismeasurement with just over half applicable for differential and non-differential mismeasurement. Also, more than half of the software tools implement a QBA for misclassification of a binary variable. Although most software tools implemented a deterministic bias analysis, only six (40%) of these tools included features to allow the user to perform a multidimensional bias analysis. Most tools provided usage examples, but documentation quality varied from minimal to extensive. While several tools offered comprehensive guidance, including tutorials and vignettes, others provided only brief descriptions of inputs and outputs. Just over half of the tools implemented all the steps of a multidimensional or probabilistic QBA for the user, but a subset required specialist QBA knowledge, such as understanding Bayesian priors or manually implementing a multidimensional bias analysis.

### Remarks on review limitations and future work

Our review did not assess the extent to which each tool is used in practice. While citation counts or software download statistics (e.g., using the R package *cranlogs*) could offer useful proxies for uptake, such metrics are not consistently available across different environments. Future work could explore quantifying the uptake of QBA tools in applied research and identifying factors associated with broader usage.

This review did not set out to evaluate the performance of the software tools or verify their outputs and so we cannot comment on their runtime behaviour. Tools which failed to produce outputs due to unhandled errors were excluded. However, we note that for some probabilistic QBAs, particularly those addressing misclassification, admissible bias-adjusted results may not always be obtainable depending on the combination of input assumptions. In such cases, some tools (e.g., R package *episensr*) are designed to filter out inadmissible results, providing informative error messages. Other tools may not include such error handling or may return non-specific error messages. This highlights an important practical consideration for users: the interpretability and robustness of software feedback can vary and may impact the usability of tools in real-world settings.

Although the focus of this review is on software implementation, we also acknowledge that another potential barrier to wider uptake of QBA is the challenge of specifying plausible bias parameter values and priors when they are not clearly identifiable from existing literature or validation data [15].

While these issues lie outside the scope of our review, applied examples that demonstrate not only the use of the software but also how plausible parameter values and priors might be selected could help reduce both barriers. We suggest that future works could build on this review by including such examples, clarifying in which cases each tool is most appropriate and highlighting differences in error handling and user support features.

Another area for expansion of this work would be broadening the scope of our searches. A limitation of this review is that we restricted our software repository searches to R packages available on CRAN and Stata commands available via the SSC archive or the *net* command. As such, we may have missed some tools available only on platforms such as GitHub or implemented in other programming environments such as Python, and without accompanying publications. These tools could be of interest to computationally oriented researchers, but may be unlikely to be widely adopted in applied epidemiological settings without mention in a formal publication or inclusion in repositories.

We also restricted our publication search to epidemiology, statistics, and health journals. This may have excluded software from other disciplines that could be applicable to health research. The numbers of these tools would likely be small, however, as different fields face very different complications and considerations to health research. Further, it is unlikely that health researchers would look outside of their field in order to find tools for use. Conducting similar reviews in domains such as psychology, engineering, or computational biology could increase awareness of potentially useful tools across disciplines.

Additionally, as discussed in Grey literature section, our review focused on tools developed or updated between 2014 and 2024, which may have led to the exclusion of older software still in use. This time period was chosen to prioritise tools likely to remain supported and aligned with current statistical practice. However, useful legacy tools may have been missed. While Table 2 provides a brief overview of additional tools not captured in our formal search, including some which fell outside of this time period (e.g., popular Stata command *episens*), we did not conduct a full evaluation of these tools. This reflects a decision to focus on tools that met our inclusion criteria; however, we acknowledge that some of these additional tools may still be useful in practice.

Future work could build on our review by systematically identifying and reviewing in detail tools that fall outside standard dissemination channels or have not been recently updated.

### Remarks on implications for QBA uptake

Although previous work has suggested that implementation challenges in QBA have largely been addressed [21], our findings indicate significant gaps remain. Existing tools rarely support QBA for categorical variables with more than two levels, and few address measurement error in continuous variables beyond classical error models. Further, many of the tools reviewed were designed for specific use cases, often allowing only a single data type or a single type of bias analysis. A notable proportion of the tools only handle non-differential mismeasurement, despite growing emphasis in the literature that differential mismeasurement is common and may introduce complex bias in real-world studies [3, 4, 8, 77]. Tools capable of addressing differential mismeasurement may therefore be of greater practical value, particularly in applied epidemiologic research. Developing or expanding software to cover these scenarios, and to handle multiple potential mismeasurement types, would improve accessibility and could lead to greater uptake by applied researchers.

While our formal search identified tools implemented in R, Stata and web environments, none were implemented in SAS (see Table 2 for details of some SAS macros not captured by our formal search strategy). Given the use of SAS in many organisations (such as government agencies, healthcare institutions, and other applied research settings), this gap in platform availability may limit the accessibility of QBA tools to some users. Increasing software availability across platforms could support broader adoption in applied research contexts.

In addition to gaps in available methods and environments, level of documentation may pose a barrier to effective tool use. While we assessed documentation quality and the level of QBA knowledge required separately, these aspects of tools are likely to be related. More extensive, good quality documentation may help to lessen the need for specialist expertise by supporting learning and correct use, while minimal documentation can make a tool inaccessible even if specialist knowledge is not required. This suggests that improving documentation could make existing tools more accessible to a broader range of researchers, especially those less familiar with QBA.

Further, although all tools met our definition of “software” by including at least minimal documentation, we found that documentation often did not explicitly state key assumptions about the data. For example, despite eight tools allowing multiple variables to be simultaneously measured with error, only R package *episensr* explicitly stated that errors were required to be independent. The lack of clear statements about underlying assumptions forces researchers to rely on prior methodological knowledge or manual code inspection, increasing the potential risk of misuse or misinterpretation. This not only exacerbates the broader issue of unacknowledged dependent error in epidemiological studies [78] but may also create a barrier to the adoption of tools by applied researchers. Addressing these gaps by explicitly stating assumptions and tool limitations in documentation could facilitate wider adoption and correct application of QBA methods.

The substantial number of tools in Table 2 that were not identified through our formal search highlights the challenge for applied researchers in discovering relevant QBA software. Many of these tools would be difficult for researchers unfamiliar with mismeasurement and quantitative bias analysis to discover, reinforcing the need for greater visibility of tools. Publishing software tools in widely recognized repositories, maintaining clear documentation of updates and expansions, and encouraging researchers to cite software in their outputs would help bridge this gap.

## Conclusions

Our review highlights an increase in the number of software tools for QBA to mismeasurement but also reveals important gaps that may limit their accessibility and applicability. While many tools support common analyses and provide extensive documentation, others lack clarity on key assumptions, require specialist knowledge, or are restricted to specific use cases. There is a lack of tools for handling misclassification of categorical variables and for addressing non-classical measurement error in continuous variables. Improved documentation, broader methodological coverage, and increased visibility through publication in software journals and good citation practices could enhance the usability and adoption of these tools. Future efforts should focus on developing more comprehensive software tools and ensuring that researchers can easily identify and apply appropriate programs for addressing mismeasurement in their studies.

## Supplementary Information.

Additional file 1: PRISMA-ScR Checklist. Filled PRISMA-ScR checklist for our scoping review methodology.

Additional file 2: CRAN Search Code. R code used to conduct the CRAN search stage.

Additional file 3: Stata Search Code. Stata code used to conduct the search of the Stata manual, Stata journal and user-written code.

## Data Availability

No datasets were generated or analysed during the current study.

## Footnotes

1 Although agreement-based metrics such as Cohen’s $\kappa$ [22] are sometimes reported for categorical variables, they are not suitable for use as bias parameters in QBA, since they measure agreement rather than the relationship between observed and true values.

2 Note that differential misclassification would require separate parameter values for different subgroups, such as cases and controls within a case-control study.

3 One of these tools, *Short code* [34, 35], is discussed further in Grey literature section and included in Table 2, due to its prominence in core QBA literature [13].

4 We note here that the R package *EValue* has a corresponding Stata implementation *evalue* [60] and web tool [61, 62]. At the time of writing, neither of these tools implement a QBA to mismeasured variables; instead, they focus on bias due to unmeasured confounding, so we have not included them here. See [14] for further details on these tools for QBA to unmeasured confounding. The web tool *SAMBA-EHR* also exists alongside R package *SAMBA* [63]. This R package does not implement a QBA to mismeasured variables and so has not been reviewed here.

5 The R package *multibias* does not perform a probabilistic QBA, but does provide guidance and example code demonstrating how a researcher can manually implement a probabilistic QBA using the tool. However, *multibias web tool* provides a function which performs a probabilistic QBA.
